# Increased endotoxin activity is associated with clinical deterioration in moderate-severity emergency department sepsis patients: a pilot study

**DOI:** 10.1186/cc11789

**Published:** 2012-11-14

**Authors:** R Arnold, C Schorr, S Trzeciak, RP Dellinger

**Affiliations:** 1 Cooper University Hospital, Camden, NJ, USA

## Background

Increasing levels of endotoxin activity are associated with increasing mortality in ICU patients. The removal of endotoxin through polymyxin B hemoperfusion has been shown to decrease organ dysfunction and reduce mortality in septic shock patients. Our objective was to determine the ability of endotoxin measurement and its change over 24 hours in normotensive sepsis patients to predict clinical deterioration.

## Methods

A prospective observational study in a single-center urban, academic medical center involving adult ED patients with suspected infection admitted to a medical floor and receiving i.v. antibiotics with either elevated lactate (2.0 to 3.9 mM) or transient hypotension (sBP <90 mmHg). Patients with overt shock (mechanical ventilation or vasopressor requirement), pregnancy, or acute trauma were excluded. The Endotoxin Activity Assay was measured at enrollment and again at 24 hours. Subjects were followed for the development of increased organ failure (Sequential Organ Failure Assessment (SOFA) score increase >1 point, mechanical ventilation or vasopressor utilization) within 72 hours of admission or in-hospital mortality.

## Results

We enrolled 57 patients over a 12-month period. The primary outcome was met in 63% of the cohort and 33% were transferred to the ICU from their initial medical floor admission. Patients with the outcome of increased organ failure had no difference in initial lactate, age, MAP or exposure to hypotension. Outcome patients had similar initial and 12-hour SOFA scores with higher scores at 24, 48, and 72 hours, a higher ICU transfer rate (42% vs. 19%, *P *= 0.15) and increased ICU (2 vs. 0) and hospital length of stays (13 vs. 8). Outcome patients had similar endotoxin levels at enrollment compared with those not meeting outcome (0.61 vs. 0.51, *P *= 0.075) with increased levels at 24 hours (0.73 vs. 0.47, *P *= 0.005). An increasing endotoxin level over 24 hours had a 71% specificity and 72% PPV in predicting the primary outcome. Compared with serum lactate, there was no relationship between endotoxin activity and initial serum lactate on linear regression analysis (slope = 0.077, *R*^2 ^= 0.0004).

## Conclusion

Within this moderate-severity sepsis cohort, physiologic data in the ED were unable to differentiate those with progressive organ dysfunction over 72 hours, while endotoxin levels were higher at 24 hours in this outcome group. The high specificity and positive predictive value of an increasing endotoxin level as a predictor of progressive organ dysfunction can supplement clinical decision-making to aid clinicians in identifying high-risk patients from this homogeneous population of moderate-severity sepsis patients.

